# Corneal Nerve Fiber Morphology and Biological Age in Healthy Adults

**DOI:** 10.3390/biomedicines14071517

**Published:** 2026-07-06

**Authors:** Anait S. Khalatyan, Yusef Yusef, Zoia V. Surnina, Kristina G. Sarkisova, Ekaterina A. Chizhonkova, Konstantin S. Avetisov, Khadishat K. Altemirova, Liubov V. Machekhina, Alexandra A. Melnitskaya, Irina D. Strazhesko

**Affiliations:** 1 Krasnov Research Institute of Eye Diseases, Moscow 119021, Russia; info@eyeacademy.ru (Y.Y.); medzoe@yandex.ru (Z.V.S.); kristina-sarkisova93@mail.ru (K.G.S.); ekka286@yandex.ru (E.A.C.); avetisov.k.s@gmail.com (K.S.A.); hadishka_16@mail.ru (K.K.A.); 2 Russian Gerontology Research and Clinical Centre, Pirogov Russian National Research Medical University, Moscow 129226, Russia; machehina_lv@rgnkc.ru (L.V.M.); melnickaya_aa@rgnkc.ru (A.A.M.); strazhesko_id@rgnkc.ru (I.D.S.)

**Keywords:** corneal confocal microscopy, corneal nerve fibers, biological age, PhenoAge, aging, peripheral neuroaging

## Abstract

**Background/Objectives:** Corneal confocal microscopy (CCM) quantifies subbasal corneal nerve fibers noninvasively and may inform peripheral neuroaging. PhenoAge is a validated clinical measure of biological aging linked to morbidity and mortality risk and therefore provides a geriatric-relevant index of systemic aging. We aimed to assess corneal nerve morphology in clinically healthy adults and determine whether CCM-derived parameters are associated with biological age (PhenoAge) beyond chronological age. **Methods:** Eighty-four healthy volunteers (22–89 years) underwent CCM. PhenoAge was calculated using the Levine algorithm. Associations with chronological age and PhenoAge were tested using Spearman correlations (eye-specific and participant-level mean of both eyes). Paired inter-eye differences were assessed, and linear mixed-effects models (random intercept for participant; fixed effects for age/PhenoAge and eye) were fitted. **Results:** Mean chronological age was 50.8 ± 15.5 years, and mean PhenoAge was 47.1 ± 16.3 years. No systematic inter-eye differences were detected (all *p* > 0.05). Across analyses, older age and higher PhenoAge were associated with lower main corneal nerve fiber measures, most consistently for main-fiber density. Participant-level sensitivity analysis (mean of both eyes) confirmed inverse associations of both chronological age and PhenoAge with main-fiber length and density (all *p* ≤ 0.035). In mixed-effects models, main-fiber density was associated with chronological age (β = −0.020/year, *p* = 0.032) and PhenoAge (β = −0.019/year, *p* = 0.037). **Conclusions:** CCM-derived corneal nerve morphology demonstrates aging-related patterns in clinically healthy adults. The association between PhenoAge and main-fiber density may suggest a systemic biological aging component and warrants longitudinal validation.

## 1. Introduction

Aging is accompanied by structural and functional decline of the peripheral nervous system, which contributes to reduced sensory function and impaired tissue homeostasis [1]. A central driver of systemic aging is chronic, low-grade, age-associated inflammation (“inflammaging”), a concept originally articulated by Franceschi and colleagues and now widely used to integrate immune aging with multi-morbidity risk [2]. Recent syntheses connect inflammaging to multiple biological “hallmarks of aging,” emphasizing how persistent inflammatory signaling interacts with other age-related cellular processes at the organism level [3]. Oxidative stress is another key mechanism implicated in nervous system aging, and experimental work highlights that oxidative stress can drive functional and morphological deficits relevant to peripheral nerve aging [4].

In parallel, a robust body of evidence demonstrates that regenerative capacity declines with age, including diminished repair programs in Schwann cells and slower axonal regrowth after injury [5].

The cornea, being the most densely innervated tissue in the human body, performs a number of critical functions that directly depend on the integrity of its neural apparatus [6]. Corneal nerve fibers (CNFs) play a key role in maintaining ocular homeostasis and the functional integrity of the corneal epithelium. Branches of the subbasal nerve plexus innervate the corneal epithelium and regulate the dynamic processes of autoregeneration and wound healing through the release of epitheliotropic mediators [7]. Because the cornea is densely innervated and accessible to noninvasive high-resolution imaging, it can serve as an in vivo window into small-fiber integrity and broader peripheral neuroaging processes. Corneal confocal microscopy (CCM), a form of in vivo confocal microscopy (IVCM), has revolutionized ophthalmology by providing a unique opportunity to noninvasively visualize, in real time and with near-histologic resolution, all layers of the cornea, including the subbasal nerve plexus [8]. Importantly, corneal confocal microscopy-derived nerve measures have been repeatedly proposed as quantitative biomarkers of small-fiber damage and repair across systemic conditions, reinforcing their relevance beyond ophthalmology [9,10]. CCM studies in healthy humans indicate that sub-basal corneal nerve metrics show age-associated changes, supporting the feasibility of age-stratified normative reference frameworks [11]. Finally, integrating corneal nerve morphology with measures of biological age (e.g., Phenotypic Age/PhenoAge) aligns this work with gerontology by linking tissue-level neural integrity to systemic morbidity and mortality risk [12].

Chronic inflammation associated with external impacts on the cornea contributes to the development of age-related degenerative changes and disrupts homeostatic processes and protective mechanisms [13]. However, a paradox remains: accurate interpretation of pathological changes requires a clear understanding of normal variability, with age serving as a key modifying factor. Without considering involutional (physiological) versus pathological changes in CNFs, there is a high risk of overdiagnosing neuropathy in older patients or, conversely, underestimating early signs of pathology in younger individuals. It is possible that such data will ultimately enable the development of a mathematical model for estimating a person’s biological age—a complex concept reflecting metabolic health status [14].

Thus, the aim of this study was to assess the status of corneal nerve fibers in clinically healthy adults across different age groups and to determine whether in vivo confocal microscopy-derived corneal nerve parameters are associated with biological age (PhenoAge), beyond chronological age.

## 2. Materials and Methods

### 2.1. Demographic Characteristics of Study Participants

Within the RussAge research project [15], a sample of 84 healthy volunteers (168 eyes) aged 22 to 89 years was formed. The work was carried out at the clinical base of the Russian Gerontology Research and Clinical Center of Pirogov Russian National Research Medical University. The ophthalmological part was carried out at the Krasnov Research Institute of Eye Diseases. The study protocol was approved by the Local Ethics Committee of the Russian Gerontology Research and Clinical Centre, Pirogov National Research Medical University (protocol No. 59 from 13 September 2022) and complied with the ethical principles of the Declaration of Helsinki; each participant provided written informed consent.

Inclusion criteria were age > 18 years and voluntary participation. Key exclusion criteria included systemic autoimmune and oncologic diseases, diabetes mellitus (type 1 or 2), cognitive or psychiatric disorders, moderate-to-severe chronic diseases, signs of acute intercurrent infection, C-reactive protein (CRP) concentrations > 10 mg/L, a history or presence at the time of examination of ophthalmic pathology (including corneal disease, refractive errors exceeding ±3.0 D, glaucoma, inflammatory conditions, age-related macular degeneration), previous ophthalmic interventions, and a history of contact lens wear. The CRP threshold was applied to minimize acute inflammation-related confounding.

### 2.2. Biological Age Assessment

For each participant, biological age was quantified using the Phenotypic Age (PhenoAge) algorithm introduced by Levine and colleagues (2018) [12]. PhenoAge is a geroscience-oriented composite index that integrates routine clinical chemistry and hematology measures to approximate systemic biological aging and age-related risk beyond chronological age. The calculation in our study incorporated nine standard biomarkers obtained during a comprehensive health assessment in a single accredited laboratory—serum albumin, creatinine, glucose, C-reactive protein, lymphocyte percentage, mean corpuscular volume, red cell distribution width, alkaline phosphatase, and total leukocyte count—together with chronological age, applying the published regression coefficients. The output is reported in years; values lower or higher than chronological age indicate relatively decelerated or accelerated biological aging, respectively.

To align systemic biomarker profiling with ocular imaging and support temporally coherent phenotyping, venous blood sampling was scheduled within 7 days prior to ophthalmological examination. Because acute inflammatory episodes can transiently distort aging-related biomarkers and confound inference about chronic low-grade inflammation relevant to gerontology, participants were screened for signs of intercurrent infection (e.g., fever or respiratory symptoms) on the examination day.

### 2.3. Ophthalmic Examination and Corneal Confocal Microscopy

All participants underwent a standard ophthalmic screening including refractometry, visometry, and tonometry, followed by collection of medical history data and a clinical examination by an ophthalmologist. For participants meeting the inclusion criteria described above, corneal nerve fiber assessment was performed using the HRT III (Heidelberg Retinal Tomograph III, Heidelberg Engineering GmbH, Heidelberg, Germany) with the Rostock Cornea Module. Confocal images were analyzed using Liner 1.2.S and Liner Calculate software version 1.1 [8,16]. In addition to conventional morphometric parameters, including the total length and density of the main corneal nerve fibers and their branches, two previously developed orientation-based coefficients were calculated: the anisotropy coefficient of corneal nerve fiber orientation (KΔL) and the directionality symmetry coefficient (Ksym) [17,18].

Briefly, the algorithm is based on automated recognition of corneal nerve fibers on digital confocal images. For each image point, the software estimates the probability of nerve fiber presence and the most likely angle of fiber orientation by comparing the local image region with a series of rotated model nerve fragments. The resulting angular distribution of detected nerve fibers is then summarized using a rose diagram.

KΔL characterizes the predominance of one main direction of nerve fiber orientation and is calculated from the ratio between the longest and shortest rays of the rose diagram. Higher KΔL values indicate greater orientation anisotropy, i.e., a more pronounced common direction of corneal nerve fibers. Ksym characterizes the symmetry of the angular distribution of nerve fiber orientations. In the normalized form used in the present study, Ksym approaches 1 when the distribution of nerve orientations is more symmetric and decreases when the distribution is more asymmetric. These coefficients provide quantitative descriptors of the orientation pattern and static tortuosity of the subbasal corneal nerve plexus.

### 2.4. Statistical Analysis

All analyses were performed at the participant level. Because multiple confocal frames were available per participant, repeated measurements for each parameter were averaged within each participant separately for the right eye and left eye prior to inferential analyses.

Descriptive statistics for quantitative variables are reported as mean, standard deviation (SD), minimum, maximum, median, and the first and third quartiles (Q1 and Q3). Normality of distributions was assessed using the Shapiro–Wilk test; because several variables deviated from normality, Spearman correlations were used. For age-stratified summaries, participants were grouped according to WHO age categories (18–44, 45–59, 60–74, and 75–90 years), and group means with SD and 95% confidence intervals (95% CIs) were calculated.

Inter-eye comparisons were performed using paired tests on participants with bilateral data: paired *t*-tests were applied when inter-eye differences were approximately normally distributed; otherwise, the Wilcoxon signed-rank test was used.

Associations between corneal nerve parameters and age were evaluated separately for chronological age and biological age (PhenoAge). Primary association analyses used Spearman’s rank correlation coefficient (ρ) and were conducted (i) for the right and left eyes separately and (ii) in a sensitivity analysis using participant-level metrics defined as the mean of both eyes (or a single-eye value when only one eye was available).

To further account for within-subject correlation between eyes and to obtain model-based effect estimates, linear mixed-effects models were fitted with a random intercept for participant. Models were specified separately for chronological age and for PhenoAge; eye (right/left) was included as a fixed effect to adjust for potential systematic inter-eye offsets. Regression coefficients (β) are reported as the expected change in the outcome per one-year increase in age (or PhenoAge), with 95% CIs and *p*-values.

A two-sided *p*-value < 0.05 was considered statistically significant. All computations and visualizations were performed in Python version 3.13.5, with pandas 2.2.3, scipy 1.17.0, and statsmodels 0.14.6. Document tables were assembled using openpyxl 3.1.5 and python-docx 1.2.0.

## 3. Results

### 3.1. Participant Characteristics and Descriptive Statistics

The mean chronological age of the cohort was 50.8 ± 15.5 years (range: 22–89), and the mean biological age (PhenoAge) was 47.1 ± 16.3 years (range: 17–92) (Table 1). The mean anisotropy (KΔL) and symmetry (Ksym) coefficients were 3.417 ± 0.879 and 0.920 ± 0.057 (Table 1).

### 3.2. Descriptive Statistics of Corneal Nerve Parameters

Eye-specific descriptive statistics are provided in Appendix A, Table A1 (right eye) and Table A2 (left eye). Main corneal nerve fiber metrics were broadly comparable between eyes at the descriptive level, with substantial inter-individual variability across parameters (Table A1 and Table A2).

### 3.3. Inter-Eye Comparisons

In paired comparisons among participants with bilateral data, no statistically significant inter-eye differences were detected for any corneal nerve parameter (all *p* > 0.05; Table 2), indicating that, at the group level, corneal nerve metrics were not systematically different between the right and left eyes when within-subject pairing was accounted for.

### 3.4. Associations with Chronological Age

Spearman correlation analyses demonstrated inverse associations between chronological age and key corneal nerve measures, most consistently for the main fiber metrics. In the right eye, chronological age was negatively correlated with total length of main CNFs (ρ = −0.279, *p* = 0.015) and density of main CNFs (ρ = −0.242, *p* = 0.035) (Table 3). In the left eye, correlations with chronological age were weaker and did not reach statistical significance for most parameters (Table 3).

In mixed-effects models accounting for within-subject correlation between eyes (random intercept for participant; fixed effects for age and eye), chronological age was significantly associated with density of main CNFs (β = −0.020 per year, 95% CI −0.038 to −0.002; *p* = 0.032), while the association with total length of main CNFs showed a trend toward significance (β = −0.216 per year, 95% CI −0.435 to 0.003; *p* = 0.053) (Table A3). No significant associations were observed for branch-related or combined measures in the mixed-effects framework (Table A3).

### 3.5. Associations with Biological Age

Eye-specific Spearman analyses showed that PhenoAge was inversely associated with main fiber measures in the right eye, including total length of main CNFs (ρ = −0.288, *p* = 0.012) and density of main CNFs (ρ = −0.244, *p* = 0.034) (Table 4). Associations in the left eye were not statistically significant (Table 4). Neither KΔL nor Ksym showed significant correlations with PhenoAge (Table 4).

In mixed-effects models, PhenoAge was significantly associated with density of main CNFs (β = −0.0186 per year, 95% CI −0.036 to −0.001; *p* = 0.037), whereas the association with total length of main CNFs did not reach statistical significance (β = −0.190 per year, 95% CI −0.399 to 0.019; *p* = 0.074) (Table A4). No significant associations were detected for branch-related or combined measures (Table A4).

### 3.6. Sensitivity Analysis (Participant-Level Mean of Both Eyes)

To evaluate systemic associations while minimizing laterality-related variability, we repeated correlation analyses using participant-level metrics computed as the mean of both eyes (or a single-eye value when only one eye was available). Using these participant-level measures, both chronological age and PhenoAge remained inversely associated with the main fiber metrics: for chronological age, total length of main CNFs (ρ = −0.248, *p* = 0.026) and density of main CNFs (ρ = −0.246, *p* = 0.028); for PhenoAge, total length of main CNFs (ρ = −0.236, *p* = 0.035) and density of main CNFs (ρ = −0.241, *p* = 0.031) (Table 5). Associations with branch-related and combined measures were not statistically significant (Table 5).

**Figure 1:**
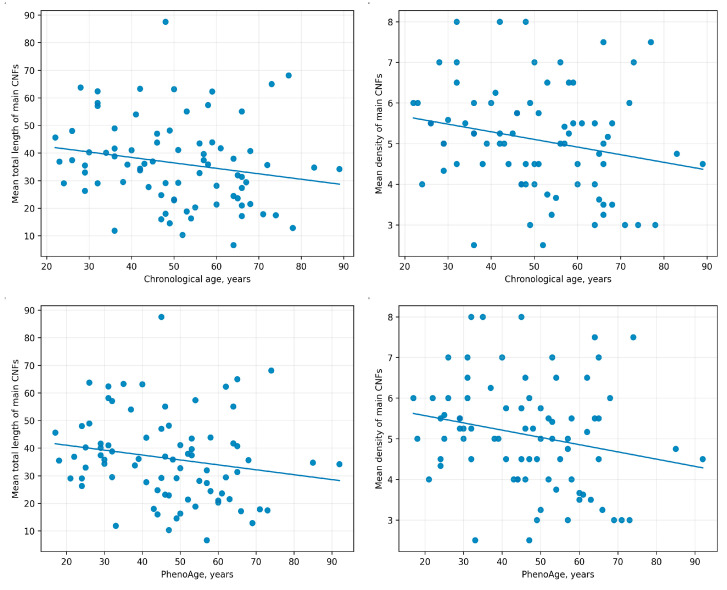
Scatter plots of participant-level mean main corneal nerve fiber parameters against chronological age and PhenoAge. The upper panels show associations of total length and density of main CNFs with chronological age, and the lower panels show associations of total length and density of main CNFs with PhenoAge. Participant-level values were calculated as the mean of both eyes or a single-eye value when only one eye was available. Trend lines are shown for visual representation only. Blue lines indicate fitted linear trend lines and are shown for visual representation only.

To provide a visual representation of the primary participant-level associations, scatter plots of the mean total length and mean density of main corneal nerve fibers against chronological age and PhenoAge were added (Figure 1). These plots illustrate the overall inverse direction of the associations, particularly for main-fiber density, which showed the most consistent relationship with both aging metrics across the participant-level and mixed-effects analyses.

### 3.7. Age-Stratified Summaries (WHO Age Categories)

**Figure 2:**
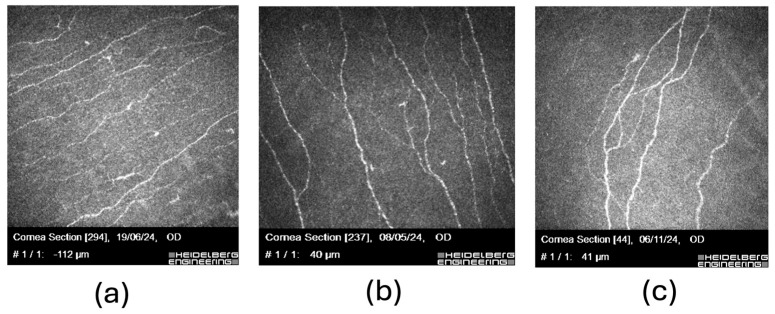
Representative corneal confocal microscopy (CCM) images of the subbasal nerve plexus in clinically healthy participants (right eye, OD) across age groups. (**a**) Young adult (born 2001): dense network of thin main nerve fibers with frequent branching; (**b**) Middle-aged adult (born 1970): reduced branching and more prominent, longitudinally oriented main fibers compared with the younger example; (**c**) Older adult (born 1946): sparse subbasal nerve pattern with fewer visible fibers and reduced branching. Images were acquired using HRT III with the Rostock Cornea Module.

Age-stratified summaries by WHO age categories for chronological age and PhenoAge, including 95% confidence intervals, are provided in Appendix A, Table A5, Table A6, Table A7 and Table A8. Across strata, mean main-fiber metrics tended to be lower in older groups, while uncertainty increased in the oldest category, consistent with smaller subgroup sizes and greater heterogeneity (See Figure 2, Table A5, Table A6, Table A7 and Table A8).

Overall, across correlation-, sensitivity-, and mixed-model analyses, the most consistent aging-related signal involved a decline in main corneal nerve fiber density, with additional supportive evidence for reduced main-fiber length.

## 4. Discussion

### 4.1. Principal Findings

In this cohort of clinically healthy adults, both chronological age and biological age (PhenoAge) were inversely associated with corneal nerve fiber morphology, with the most consistent relationships observed for the main corneal nerve fiber measures—particularly main fiber density, with supportive evidence for reduced main fiber length across analyses. Model-based analyses accounting for paired-eye data (linear mixed-effects models) confirmed a significant association between age/PhenoAge and main fiber density, and participant-level sensitivity analysis using the mean of both eyes demonstrated that the biological-age signal is present at the individual level, supporting a systemic aging-related component.

### 4.2. Comparison with Previous Studies

Our findings are broadly consistent with normative CCM studies reporting age-related reductions in subbasal nerve parameters in healthy participants, with the most reproducible signal in our data observed for main corneal nerve fiber density and supportive evidence for reduced main-fiber length. In most studies, authors report a statistically significant age-related decrease in CNF density and/or length in healthy individuals. For example, Chin et al. reported lower nerve length and density in participants older than 65 years [19]. Niederer et al. described a linear age-related decrease in density of 0.9% per year (*p* < 0.001), and Tavakoli et al. reported an annual decrease in CCM-derived nerve metrics of approximately 0.16 fibers/mm^2^ (*p* < 0.01) [11,20]. Grupcheva et al. also found an age-related decrease in nerve density (582.39 ± 327.13 μm/mm^2^ at 70 years vs. 632.35 ± 287.57 μm/mm^2^ at 25 years) [21]. In the study by Parissi et al., subbasal nerve density decreased by 0.25–0.30% per year (with a mean value of 19 mm/mm^2^) in healthy individuals [22]. Dehghani et al. also described a linear age-related decrease in CNF length (−0.05 mm/mm^2^ per year) [23].

At the same time, the literature remains heterogeneous, and some studies report weak or absent associations with age depending on acquisition regions, image selection strategy, quantification approaches, and population characteristics; this variability has been emphasized in methodological discussions of CCM use as a small-fiber biomarker [9]. In this context, our approach strengthens comparability by analyzing eye-specific associations and confirming systemic effects using participant-level sensitivity analysis (mean of both eyes) and mixed-effects models that account for paired-eye data.

Beyond chronological aging, our results add a gerontological dimension by demonstrating that a clinical biomarker-based aging measure (PhenoAge)—originally developed and validated for morbidity and mortality risk—also aligns with corneal small-fiber metrics in a healthy cohort [12]. This supports the concept that corneal nerve morphology may reflect multidomain biological aging processes rather than chronological time alone.

Nevertheless, several studies did not detect statistically significant age-related differences in CNFs among healthy individuals, including reports focusing on density, number, tortuosity [24], nerve number and beading-like changes [25], and density/length reference values in specific populations [26]. In addition, tortuosity-related parameters show inconsistent age trends across studies, with both positive [20,24] and inverse correlations reported [27], underscoring the need for standardized acquisition and analytic approaches and for careful interpretation of secondary morphology metrics.

### 4.3. Biological Interpretation and Potential Mechanisms

Age-related changes in corneal nerve morphology may reflect broader processes of peripheral neuroaging, including cumulative oxidative injury, altered neurotrophic support, and reduced regenerative capacity [5,28].

Experimental evidence links oxidative stress to age-related deficits in the peripheral nervous system, providing a plausible mechanistic substrate for gradual small-fiber structural decline over the adult lifespan [4].

In addition, “inflammaging”—chronic low-grade systemic inflammation—may contribute to neural vulnerability through persistent immune activation and tissue remodeling pathways [2].

These systemic aging processes are directly relevant to PhenoAge because the algorithm incorporates biomarkers related to inflammation (e.g., CRP), metabolic state (e.g., glucose), and hematologic/immune status (e.g., leukocyte count and lymphocyte percentage), which together capture multidomain physiological dysregulation.

Accordingly, the observed associations—most consistently involving main corneal nerve fiber density (and to a lesser extent main-fiber length)—support the interpretation that corneal small-fiber morphology may track aspects of systemic biological aging burden rather than chronological time alone. The stronger, more consistent signal for main fibers than for branches may reflect both biology and measurement. Main nerve trunks form a relatively stable structural scaffold of the subbasal nerve plexus and may therefore better reflect cumulative systemic aging burden [29,30]. Distal branches, by contrast, are more dynamic: they support epithelial trophism and local tissue repair; their quantification is more sensitive to regenerative turnover, ocular-surface conditions, frame selection, and segmentation [29,31,32]. This added variability reduces the power to detect linear branch-age associations in a modest cross-sectional cohort. One interpretation may be that aging preserves the major nerve scaffold while distal branches undergo more variable remodeling, a possible prioritization of main-trunk maintenance over peripheral branching. Given the cross-sectional design, this remains hypothesis-generating and requires longitudinal validation to establish whether trunk and branch changes are sequential or partially independent.

### 4.4. Inter-Eye Similarity, Apparent Right-Eye Predominance, and Methodological Considerations

Most normative CCM work assumes substantial interocular similarity, and several studies report no meaningful between-eye differences for key nerve parameters in healthy individuals [33]. In our dataset, paired inter-eye comparisons did not demonstrate systematic differences between right and left eyes at the group level (Table 2), supporting overall interocular similarity.

Despite this, eye-specific correlation analyses showed that associations with PhenoAge were more apparent in the right eye. This pattern may reflect a combination of sampling variation and measurement precision (e.g., image quality, sampling location, segmentation performance) and differences in missingness across eye-specific parameters, rather than true biological laterality. Importantly, participant-level sensitivity analysis using the mean of both eyes confirmed significant associations between PhenoAge and the primary main-fiber measures (Table 5), and mixed-effects models accounting for paired-eye data supported an aging-related signal for main fiber density (Table A3 and Table A4), reducing concerns that the findings are driven by laterality.

Future studies should continue to use paired/hierarchical approaches (e.g., mixed-effects or GEE models) to account for within-subject correlation and to quantify any residual inter-eye offsets when present.

### 4.5. Age-Stratified Estimates and Uncertainty in Older Groups

The WHO-based age-stratified summaries with 95% confidence intervals (Table A5, Table A6, Table A7 and Table A8) show that uncertainty increases in the oldest categories, which is expected due to smaller subgroup sizes and greater biological heterogeneity in later life [34,35].

The estimates for the oldest chronological age group should be interpreted with particular caution. This group included only four participants; therefore, the corresponding means and 95% confidence intervals are imprecise and should be regarded as descriptive rather than inferential. Accordingly, our study does not provide sufficient statistical power to draw robust or generalizable conclusions specifically for individuals aged 75–90 years. The age-stratified analysis was used primarily to illustrate the direction and variability of corneal nerve parameters across age categories, whereas the main conclusions of the study are based on continuous age and PhenoAge analyses across the full cohort.

Importantly, increased dispersion with age is not merely “noise”: heterogeneity is a characteristic feature of aging populations, reflecting divergent trajectories of resilience, subclinical disease burden, and cumulative exposures—factors that biological age measures are specifically designed to capture [36].

In this context, stratification by biological age (PhenoAge) may further accentuate between-person variability because individuals of similar chronological age can differ substantially in systemic physiological dysregulation and aging burden.

### 4.6. Clinical and Gerontological Implications

From a clinical standpoint, these data support incorporating age-aware reference interpretation when evaluating CCM-derived corneal nerve metrics—particularly main corneal nerve fiber density (and secondary main-fiber length)—to reduce the risk of over-interpreting involutional changes as pathology in older adults or overlooking early deviations in younger individuals [19].

From a gerontology perspective, the association between corneal nerve morphology and PhenoAge suggests that CCM may contribute to noninvasive phenotyping of biological aging, complementing laboratory-based aging metrics that are known to track morbidity and mortality risk [37].

This aligns with the shift in geriatrics toward function-centered frameworks such as intrinsic capacity, which conceptualizes healthy aging as a composite of physical and mental capacities; corneal small-fiber integrity may be a candidate marker within the broader “sensory/vitality” domain, although this requires direct validation [38,39].

Given that accelerated biological aging measures (including PhenoAge-based indices) have been linked to trajectories of multimorbidity in large populations, the present findings support the hypothesis that corneal nerve morphology may serve as an adjunct marker of systemic aging burden in otherwise healthy adults; however, longitudinal studies are needed to establish predictive value and clinical utility.

### 4.7. Limitations

This study has several limitations. First, its cross-sectional design precludes causal inference and assessment of within-person change in corneal nerve parameters over time. Second, the sample size was modest, which limits statistical power—particularly in the oldest age strata—and the age-stratified analyses should be considered exploratory. In particular, the oldest chronological age group (75–90 years) included only four participants; therefore, estimates for this subgroup are imprecise and should not be used to draw robust or generalizable conclusions for this demographic. Third, as with any image-based biomarker, measurement variability related to acquisition and quantification is possible; this was addressed by averaging repeated measurements and using paired-eye modeling. Finally, multiple correlated endpoints were tested; therefore, findings should be interpreted with emphasis on effect sizes and consistency across sensitivity and mixed-model analyses rather than nominal *p*-values alone.

## 5. Conclusions

In clinically healthy adults, corneal nerve morphology assessed by corneal confocal microscopy demonstrated age-related patterns, with the most consistent finding being an inverse association between age and main corneal nerve fiber density, supported by a similar (but generally weaker) signal for main-fiber length. Importantly, associations with biological age (PhenoAge) were reproduced in participant-level sensitivity analysis using the mean of both eyes and were supported by mixed-effects models accounting for paired-eye data, suggesting that these corneal small-fiber measures capture a systemic component of biological aging beyond chronological age. Taken together, our results indicate that CCM-derived corneal nerve metrics—particularly main-fiber density—may serve as a practical, noninvasive adjunct for biological aging phenotyping in research settings, while longitudinal and outcome-based studies are needed to establish predictive value and clinical utility.

## Figures and Tables

**Table 1:** Descriptive statistics (overall cohort).

Parameter	Mean	SD	Min	Max	Median	Q1	Q3
Chronological age	50.798	15.501	22	89	50	38.75	64
Biological age	47.071	16.285	17	92	47	32	58.5
KΔL	3.417	0.879	1.8	5.87	3.315	2.77	3.765
Ksym	0.92	0.057	0.555	1	0.93	0.9	0.95

**Table 2:** Paired inter-eye comparisons of corneal nerve parameters.

Parameter	*n* Pairs	*p*-Value	Test
Total length of main CNFs	68	0.35	Wilcoxon signed-rank
Density of main CNFs	69	0.555	Paired *t*-test
Total length of CNF branches	67	0.314	Paired *t*-test
Density of CNF branches	67	0.469	Paired *t*-test
Total length (main CNFs + branches)	64	0.47	Paired *t*-test
Combined density (main CNFs + branches)	65	0.692	Paired *t*-test

**Table 3:** Spearman correlations with chronological age (eye-specific).

Parameter	Eye	*n*	Spearman’s ρ	*p*-Value
KΔL		81	−0.114	0.311
Ksym		81	−0.094	0.405
Total length of main CNFs	Right eye	75	−0.279	0.015
Total length of main CNFs	Left eye	73	−0.166	0.159
Density of main CNFs	Right eye	76	−0.242	0.035
Density of main CNFs	Left eye	73	−0.198	0.093
Total length of CNF branches	Right eye	75	−0.052	0.657
Total length of CNF branches	Left eye	72	−0.094	0.434
Density of CNF branches	Right eye	75	−0.085	0.467
Density of CNF branches	Left eye	72	−0.047	0.696
Total length (main CNFs + branches)	Right eye	73	−0.218	0.064
Total length (main CNFs + branches)	Left eye	71	−0.162	0.178
Combined density (main CNFs + branches)	Right eye	74	−0.181	0.124
Combined density (main CNFs + branches)	Left eye	71	−0.108	0.372

**Table 4:** Spearman correlations with biological age (eye-specific).

Parameter	Eye	*n*	Spearman’s ρ	*p*-Value
KΔL		81	−0.106	0.346
Ksym		81	−0.083	0.462
Total length of main CNFs	Right eye	75	−0.288	0.012
Total length of main CNFs	Left eye	73	−0.147	0.213
Density of main CNFs	Right eye	76	−0.244	0.034
Density of main CNFs	Left eye	73	−0.189	0.109
Total length of CNF branches	Right eye	75	−0.051	0.664
Total length of CNF branches	Left eye	72	−0.087	0.466
Density of CNF branches	Right eye	75	−0.089	0.446
Density of CNF branches	Left eye	72	−0.042	0.727
Total length (main CNFs + branches)	Right eye	73	−0.24	0.041
Total length (main CNFs + branches)	Left eye	71	−0.154	0.201
Combined density (main CNFs + branches)	Right eye	74	−0.192	0.102
Combined density (main CNFs + branches)	Left eye	71	−0.118	0.326

**Table 5:** Sensitivity analysis (participant-level mean of both eyes): Spearman correlations with chronological age (Chrono) and PhenoAge.

Parameter	*n* (Chrono)	ρ (Chrono)	*p* (Chrono)	*n* (PhenoAge)	ρ (PhenoAge)	*p* (PhenoAge)
Total length of main CNFs	80	−0.248	0.026	80	−0.236	0.035
Density of main CNFs	80	−0.246	0.028	80	−0.241	0.031
Total length of CNF branches	80	−0.083	0.466	80	−0.069	0.542
Density of CNF branches	80	−0.069	0.542	80	−0.064	0.573
Total length (main CNFs + branches)	80	−0.192	0.088	80	−0.192	0.089
Combined density (main CNFs + branches)	80	−0.166	0.141	80	−0.17	0.131

## Data Availability

The original contributions presented in this study are included in the article and Appendix A. Further inquiries can be directed to the corresponding author.

## Abbreviations

The following abbreviations are used in this manuscript:

CI	confidence interval
CCM	corneal confocal microscopy
CNF(s)	corneal nerve fiber(s)
CRP	C-reactive protein
GEE	generalized estimating equations
HRT III	Heidelberg Retinal Tomograph III
IVCM	in vivo confocal microscopy
KΔL	anisotropy coefficient of corneal nerve orientation
Ksym	symmetry coefficient of corneal nerve orientation
PNS	peripheral nervous system
PhenoAge	phenotypic age
SD	standard deviation
WHO	World Health Organization

## Appendix A

**Table A1:** Descriptive statistics for corneal nerve parameters (right eye).

Parameter	Mean	SD	Min	Max	Median	Q1	Q3	*n*
Total length of main CNFs	35.463	17.769	7.41	87.06	33.67	22.862	44.16	75
Density of main CNFs	5.044	1.56	2	9	5	4	6	76
Total length of CNF branches	10.46	8.307	0.68	41.81	8.07	5.095	15.08	75
Density of CNF branches	4.12	1.665	1	9	4	3	5	75
Total length (main CNFs + branches)	45.412	21.876	8.74	109.51	42.26	32.08	55.58	73
Combined density (main CNFs + branches)	17.577	6.027	2	32	18	15	21	74

**Table A2:** Descriptive statistics for corneal nerve parameters (left eye).

Parameter	Mean	SD	Min	Max	Median	Q1	Q3	*n*
Total length of main CNFs	36.793	19.711	2.27	102.43	36.355	23.635	47.085	73
Density of main CNFs	5.055	1.6	1	9	5	4	6	73
Total length of CNF branches	11.655	8.196	1.62	33.95	9.16	5.546	15.87	72
Density of CNF branches	4.285	1.809	1	9	4	3	6	72
Total length (main CNFs + branches)	47.397	22.999	4.42	111.53	47.69	28.055	60.212	71
Combined density (main CNFs + branches)	17.721	6.773	1	28	19	14	22	71

**Table A3:** Linear mixed-effects models for corneal nerve parameters vs. chronological age (fixed effects: age and eye; random intercept: participant).

Parameter	Participants	Observations	β per Year	SE	95% CI (L)	95% CI (U)	*p*-Value
Total length of main CNFs	80	148	−0.216	0.112	−0.435	0.003	0.053
Density of main CNFs	80	149	−0.02	0.009	−0.038	−0.002	0.032
Total length of CNF branches	80	147	0.02	0.051	−0.081	0.12	0.699
Density of CNF branches	80	147	−0.004	0.011	−0.026	0.017	0.678
Total length (main CNFs + branches)	80	144	−0.174	0.141	−0.451	0.103	0.218
Combined density (main CNFs + branches)	80	145	−0.045	0.041	−0.125	0.035	0.272

**Table A4:** Linear mixed-effects models for corneal nerve parameters vs. biological age (fixed effects: biological age and eye; random intercept: participant).

Parameter	Participants	Observations	β per Year	SE	95% CI (L)	95% CI (U)	*p*-Value
Total length of main CNFs	80	148	−0.19	0.107	−0.399	0.019	0.074
Density of main CNFs	80	149	−0.019	0.009	−0.036	−0.001	0.037
Total length of CNF branches	80	147	0.037	0.049	−0.059	0.132	0.451
Density of CNF branches	80	147	−0.001	0.01	−0.022	0.019	0.897
Total length (main CNFs + branches)	80	144	−0.15	0.135	−0.414	0.114	0.266
Combined density (main CNFs + branches)	80	145	−0.042	0.039	−0.118	0.033	0.273

Note: Models were fitted using mixed-effects regression with participant-level random intercepts to account for within-subject correlation between eyes. Eye (right/left) was included as a fixed effect to adjust for potential systematic offsets. β represents the expected change in the parameter per one-year increase in age/PhenoAge.

**Table A5:** WHO age categories (chronological age): right eye (mean ± SD, range, and 95% CI).

WHO Group	Eye	Parameter	*n*	Mean	SD	Min	Max	95% CI (Lower)	95% CI (Upper)
18–44	Right eye	Total length of main CNFs	28	41.451	16.736	15.74	87.06	34.961	47.941
18–44	Right eye	Density of main CNFs	28	5.548	1.418	3	8	4.998	6.098
18–44	Right eye	Total length of CNF branches	28	11.843	8.594	0.68	38	8.511	15.175
18–44	Right eye	Density of CNF branches	28	4.554	1.835	1	9	3.842	5.265
18–44	Right eye	Total length (main CNFs + branches)	27	51.288	18.971	16.43	109.51	43.783	58.793
18–44	Right eye	Combined density (main CNFs + branches)	27	19.506	5.405	4	32	17.368	21.644
45–59	Right eye	Total length of main CNFs	27	32.998	17.812	7.41	85.14	25.952	40.044
45–59	Right eye	Density of main CNFs	27	4.778	1.463	2	8	4.199	5.357
45–59	Right eye	Total length of CNF branches	27	8.988	5.785	0.875	25.475	6.7	11.277
45–59	Right eye	Density of CNF branches	27	3.778	1.375	1	6.5	3.234	4.322
45–59	Right eye	Total length (main CNFs + branches)	26	42.587	20.258	8.74	94.02	34.405	50.77
45–59	Right eye	Combined density (main CNFs + branches)	27	16.556	5.808	2	25	14.258	18.853
60–74	Right eye	Total length of main CNFs	16	26.593	13.631	8.39	63.74	19.329	33.856
60–74	Right eye	Density of main CNFs	17	4.5	1.668	2	9	3.643	5.357
60–74	Right eye	Total length of CNF branches	16	7.782	7.462	1.33	30.82	3.806	11.759
60–74	Right eye	Density of CNF branches	16	3.5	1.39	2	7	2.759	4.241
60–74	Right eye	Total length (main CNFs + branches)	16	34.673	20.8	9.72	99.37	23.59	45.757
60–74	Right eye	Combined density (main CNFs + branches)	16	14.688	5.885	2	28	11.552	17.823
75–90	Right eye	Total length of main CNFs	4	45.659	25.318	11.96	72.5	5.372	85.946
75–90	Right eye	Density of main CNFs	4	5.625	2.056	3	8	2.353	8.897
75–90	Right eye	Total length of CNF branches	4	21.415	15.283	6.87	41.81	−2.903	45.733
75–90	Right eye	Density of CNF branches	4	5.875	1.75	4	8	3.09	8.66
75–90	Right eye	Total length (main CNFs + branches)	4	67.069	34.097	18.82	95.56	12.813	121.324
75–90	Right eye	Combined density (main CNFs + branches)	4	23	6.218	14	28	13.105	32.895

**Table A6:** WHO age categories (chronological age): left eye (mean ± SD, range, and 95% CI).

WHO Group	Eye	Parameter	*n*	Mean	SD	Min	Max	95% CI (Lower)	95% CI (Upper)
18–44	Left eye	Total length of main CNFs	26	39.152	17.234	5.74	71.28	32.192	46.113
18–44	Left eye	Density of main CNFs	26	5.327	1.523	2	8	4.712	5.942
18–44	Left eye	Total length of CNF branches	26	11.947	7.382	1.74	25.31	8.966	14.929
18–44	Left eye	Density of CNF branches	26	4.423	1.963	1	9	3.63	5.216
18–44	Left eye	Total length (main CNFs + branches)	25	50.847	23.112	7.48	94.515	41.307	60.387
18–44	Left eye	Combined density (main CNFs + branches)	25	18.4	7.194	2	28	15.431	21.369
45–59	Left eye	Total length of main CNFs	25	39.916	23.279	13.235	102.43	30.307	49.525
45–59	Left eye	Density of main CNFs	25	5.307	1.601	3	9	4.646	5.968
45–59	Left eye	Total length of CNF branches	24	11.887	8.015	1.62	30.215	8.502	15.271
45–59	Left eye	Density of CNF branches	24	4.243	1.836	1	8	3.468	5.018
45–59	Left eye	Total length (main CNFs + branches)	24	49.199	24.028	14.855	111.53	39.053	59.345
45–59	Left eye	Combined density (main CNFs + branches)	24	17.986	6.579	4	28	15.208	20.764
60–74	Left eye	Total length of main CNFs	18	30.719	16.449	2.27	65.01	22.539	38.899
60–74	Left eye	Density of main CNFs	18	4.491	1.575	1	7	3.707	5.274
60–74	Left eye	Total length of CNF branches	18	7.984	5.41	2.15	26.455	5.293	10.674
60–74	Left eye	Density of CNF branches	18	3.787	1.496	1.5	7.5	3.043	4.531
60–74	Left eye	Total length (main CNFs + branches)	18	38.705	19.752	4.42	75.505	28.882	48.527
60–74	Left eye	Combined density (main CNFs + branches)	18	15.861	6.808	1	27	12.476	19.247
75–90	Left eye	Total length of main CNFs	4	29.264	23.538	13.71	63.725	−8.191	66.718
75–90	Left eye	Density of main CNFs	4	4.25	1.893	3	7	1.238	7.262
75–90	Left eye	Total length of CNF branches	4	24.881	12.736	6.83	33.95	4.616	45.146
75–90	Left eye	Density of CNF branches	4	5.875	1.315	4	7	3.783	7.967
75–90	Left eye	Total length (main CNFs + branches)	4	54.144	28.156	20.53	88.725	9.342	98.946
75–90	Left eye	Combined density (main CNFs + branches)	4	20.25	5.315	14	27	11.793	28.707

**Table A7:** WHO age categories (biological age): right eye (mean ± SD, range, and 95% CI).

WHO Group	Eye	Parameter	*n*	Mean	SD	Min	Max	95% CI (Lower)	95% CI (Upper)
18–44	Right eye	Total length of main CNFs	31	40.706	17.659	14.49	87.06	34.228	47.183
18–44	Right eye	Density of main CNFs	31	5.478	1.482	3	8	4.935	6.022
18–44	Right eye	Total length of CNF branches	31	11.042	8.453	0.68	38	7.942	14.143
18–44	Right eye	Density of CNF branches	31	4.435	1.785	1	9	3.781	5.09
18–44	Right eye	Total length (main CNFs + branches)	30	49.89	19.794	16.43	109.51	42.499	57.282
18–44	Right eye	Combined density (main CNFs + branches)	30	19.189	5.348	4	32	17.192	21.186
45–59	Right eye	Total length of main CNFs	26	32.194	16.279	7.41	85.14	25.619	38.769
45–59	Right eye	Density of main CNFs	26	4.731	1.328	2	8	4.194	5.267
45–59	Right eye	Total length of CNF branches	26	8.671	6.035	0.875	25.475	6.233	11.109
45–59	Right eye	Density of CNF branches	26	3.654	1.405	1	6.5	3.086	4.222
45–59	Right eye	Total length (main CNFs + branches)	25	41.445	19.468	8.74	94.02	33.409	49.481
45–59	Right eye	Combined density (main CNFs + branches)	26	16.192	5.743	2	25	13.873	18.512
60–74	Right eye	Total length of main CNFs	15	27.899	18.722	8.39	72.5	17.531	38.267
60–74	Right eye	Density of main CNFs	16	4.562	1.974	2	9	3.511	5.614
60–74	Right eye	Total length of CNF branches	15	9.311	7.454	1.33	30.82	5.184	13.439
60–74	Right eye	Density of CNF branches	15	3.833	1.447	2	7	3.032	4.635
60–74	Right eye	Total length (main CNFs + branches)	15	37.528	25.258	9.72	99.37	23.541	51.516
60–74	Right eye	Combined density (main CNFs + branches)	15	15.4	6.749	2	28	11.663	19.137
75–90	Right eye	Total length of main CNFs	1	44.425		44.425	44.425		
75–90	Right eye	Density of main CNFs	1	5.5		5.5	5.5		
75–90	Right eye	Total length of CNF branches	1	23.785		23.785	23.785		
75–90	Right eye	Density of CNF branches	1	6.5		6.5	6.5		
75–90	Right eye	Total length (main CNFs + branches)	1	68.205		68.205	68.205		
75–90	Right eye	Combined density (main CNFs + branches)	1	24		24	24		

**Table A8:** WHO age categories (biological age): left eye (mean ± SD, range, and 95% CI).

WHO Group	Eye	Parameter	*n*	Mean	SD	Min	Max	95% CI (Lower)	95% CI (Upper)
18–44	Left eye	Total length of main CNFs	28	37.533	17.178	5.74	71.28	30.872	44.194
18–44	Left eye	Density of main CNFs	28	5.232	1.481	2	8	4.658	5.807
18–44	Left eye	Total length of CNF branches	28	11.184	7.222	1.74	25.31	8.384	13.984
18–44	Left eye	Density of CNF branches	28	4.339	1.986	1	9	3.569	5.109
18–44	Left eye	Total length (main CNFs + branches)	27	48.394	22.993	7.48	94.515	39.298	57.49
18–44	Left eye	Combined density (main CNFs + branches)	27	18.037	7.085	2	28	15.234	20.84
45–59	Left eye	Total length of main CNFs	25	37.49	19.453	6.61	90.01	29.461	45.52
45–59	Left eye	Density of main CNFs	25	5.173	1.434	3	8	4.581	5.765
45–59	Left eye	Total length of CNF branches	25	11.718	7.651	1.62	30.215	8.56	14.876
45–59	Left eye	Density of CNF branches	25	4.247	1.724	1	8	3.535	4.958
45–59	Left eye	Total length (main CNFs + branches)	25	49.21	23.276	14.61	111.53	39.602	58.818
45–59	Left eye	Combined density (main CNFs + branches)	25	18.2	6.238	4	28	15.625	20.775
60–74	Left eye	Total length of main CNFs	17	35.94	25.183	2.27	102.43	22.992	48.888
60–74	Left eye	Density of main CNFs	17	4.716	2.035	1	9	3.669	5.762
60–74	Left eye	Total length of CNF branches	16	9.028	7.164	2.15	26.455	5.211	12.845
60–74	Left eye	Density of CNF branches	16	3.958	1.677	1.5	7.5	3.065	4.852
60–74	Left eye	Total length (main CNFs + branches)	16	40.814	24.388	4.42	88.725	27.818	53.809
60–74	Left eye	Combined density (main CNFs + branches)	16	15.948	7.713	1	27	11.838	20.058
75–90	Left eye	Total length of main CNFs	1	25		25	25		
75–90	Left eye	Density of main CNFs	1	4		4	4		
75–90	Left eye	Total length of CNF branches	1	33.74		33.74	33.74		
75–90	Left eye	Density of CNF branches	1	6		6	6		
75–90	Left eye	Total length (main CNFs + branches)	1	58.76		58.76	58.76		
75–90	Left eye	Combined density (main CNFs + branches)	1	20		20	20		

Note: The oldest age category included few participants; therefore, means and 95% confidence intervals are imprecise and may extend beyond plausible bounds due to sampling variability.
